# Supplementary figures and images for: Bioactivity of compounds secreted by symbiont bacteria of Nudibranchs from Indonesia
Source: PeerJ. 2020 Jan 2;8:e8093. doi: 10.7717/peerj.8093 (PMC6942679; doi:10.7717/peerj.8093)

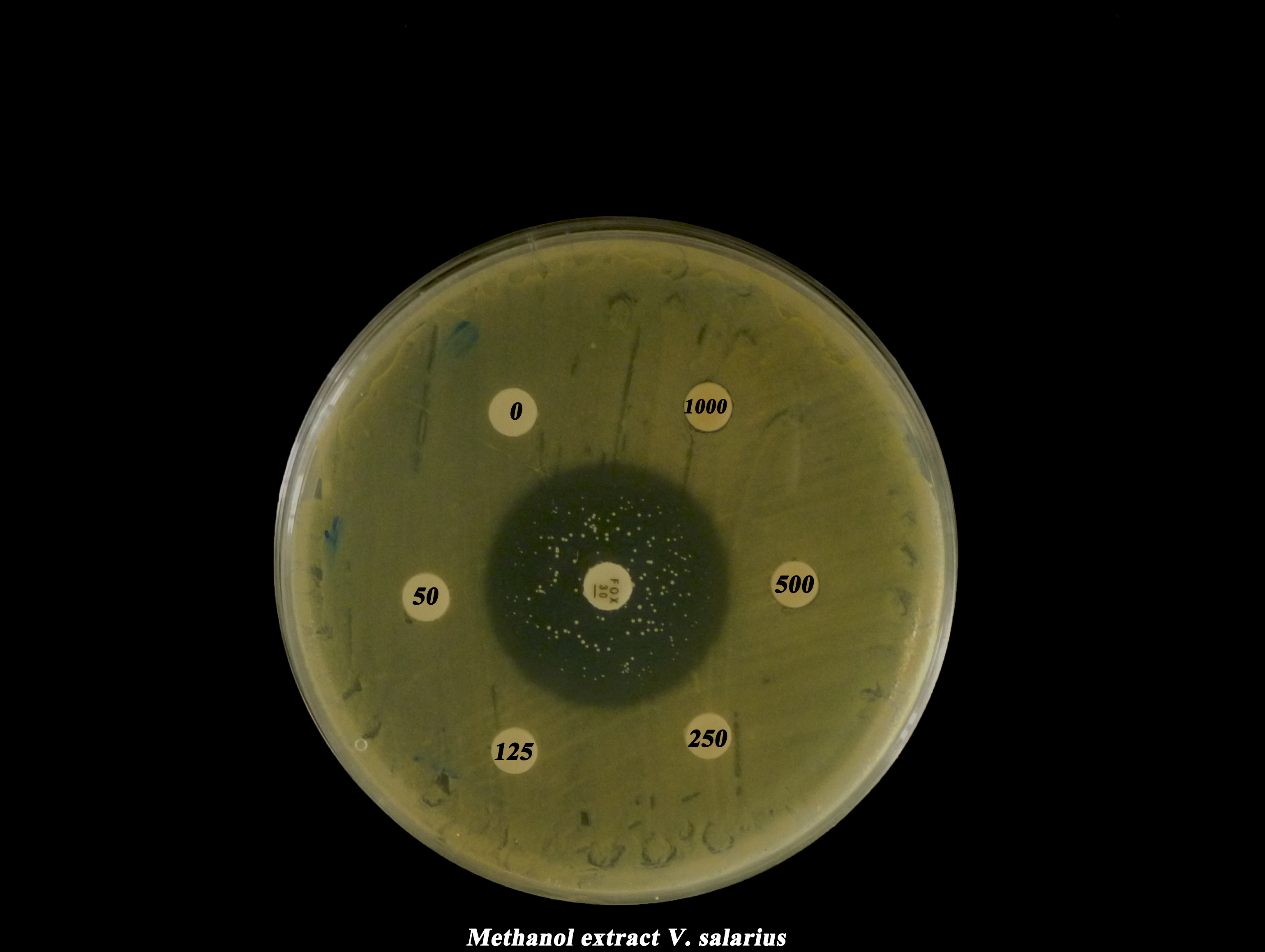

Supplement: Supplemental Information 1 [file peerj-08-8093-s001.jpg]

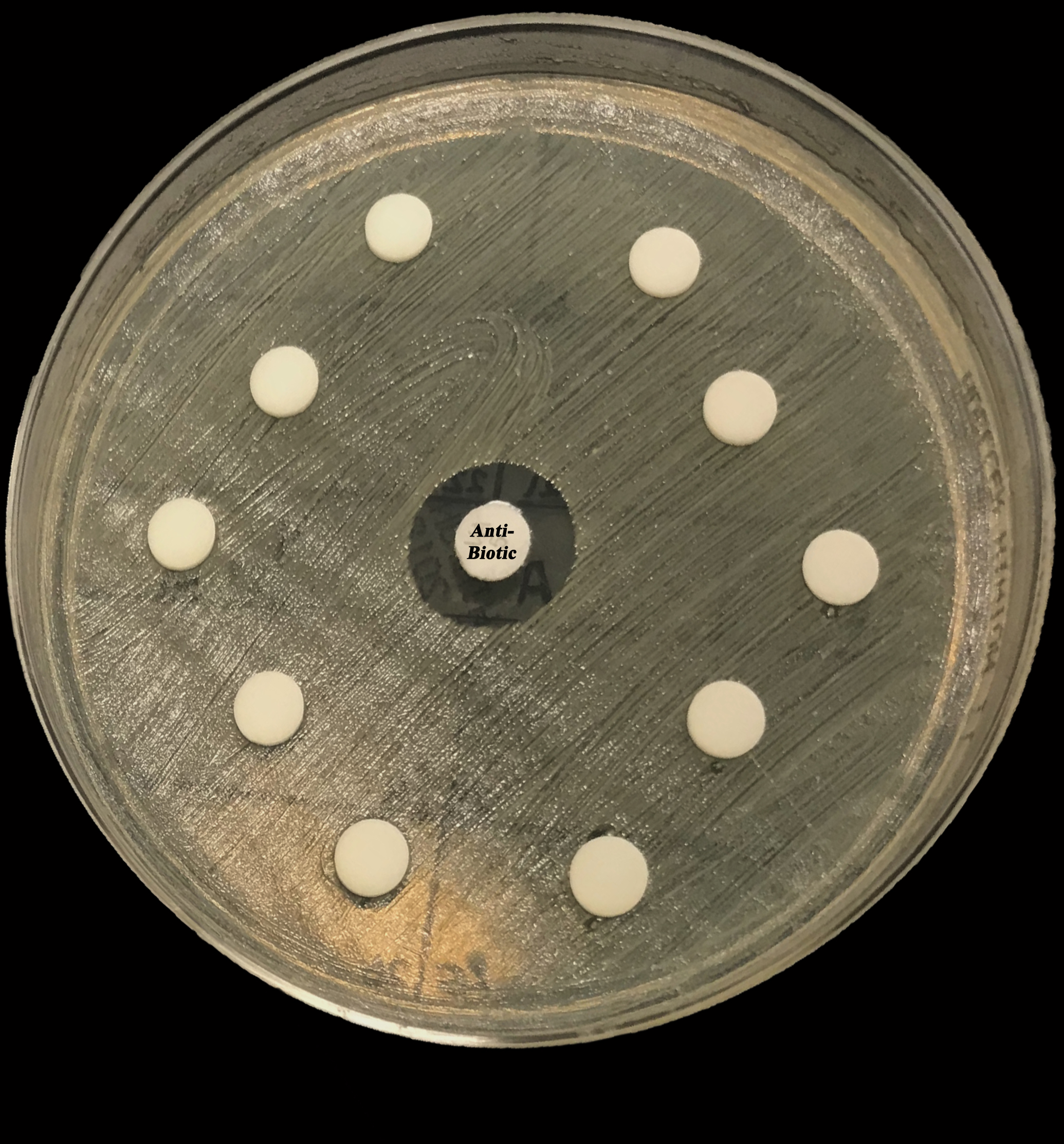

Supplement: Supplemental Information 2 [file peerj-08-8093-s002.jpg]

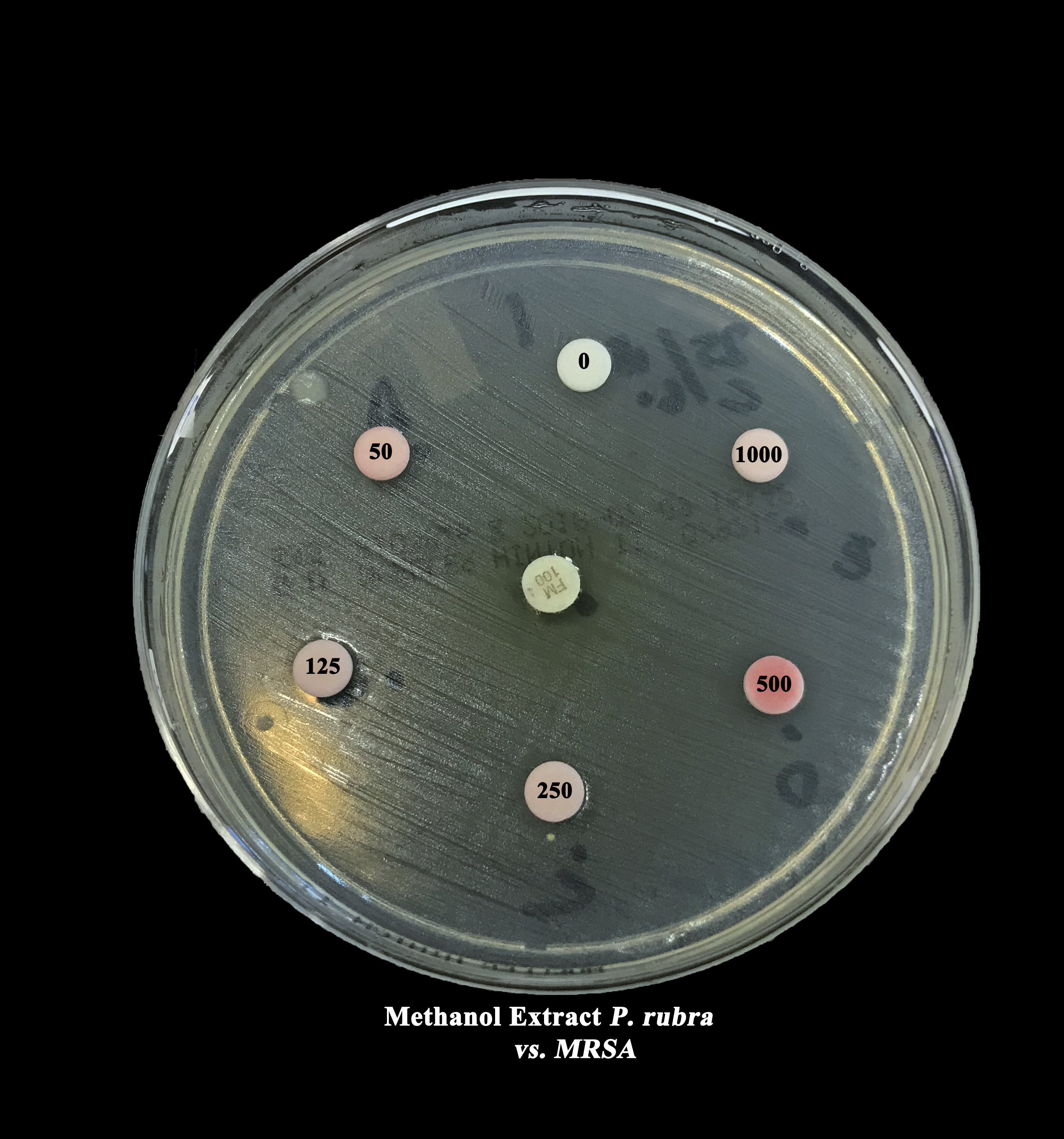

Supplement: Supplemental Information 3 [file peerj-08-8093-s003.jpg]

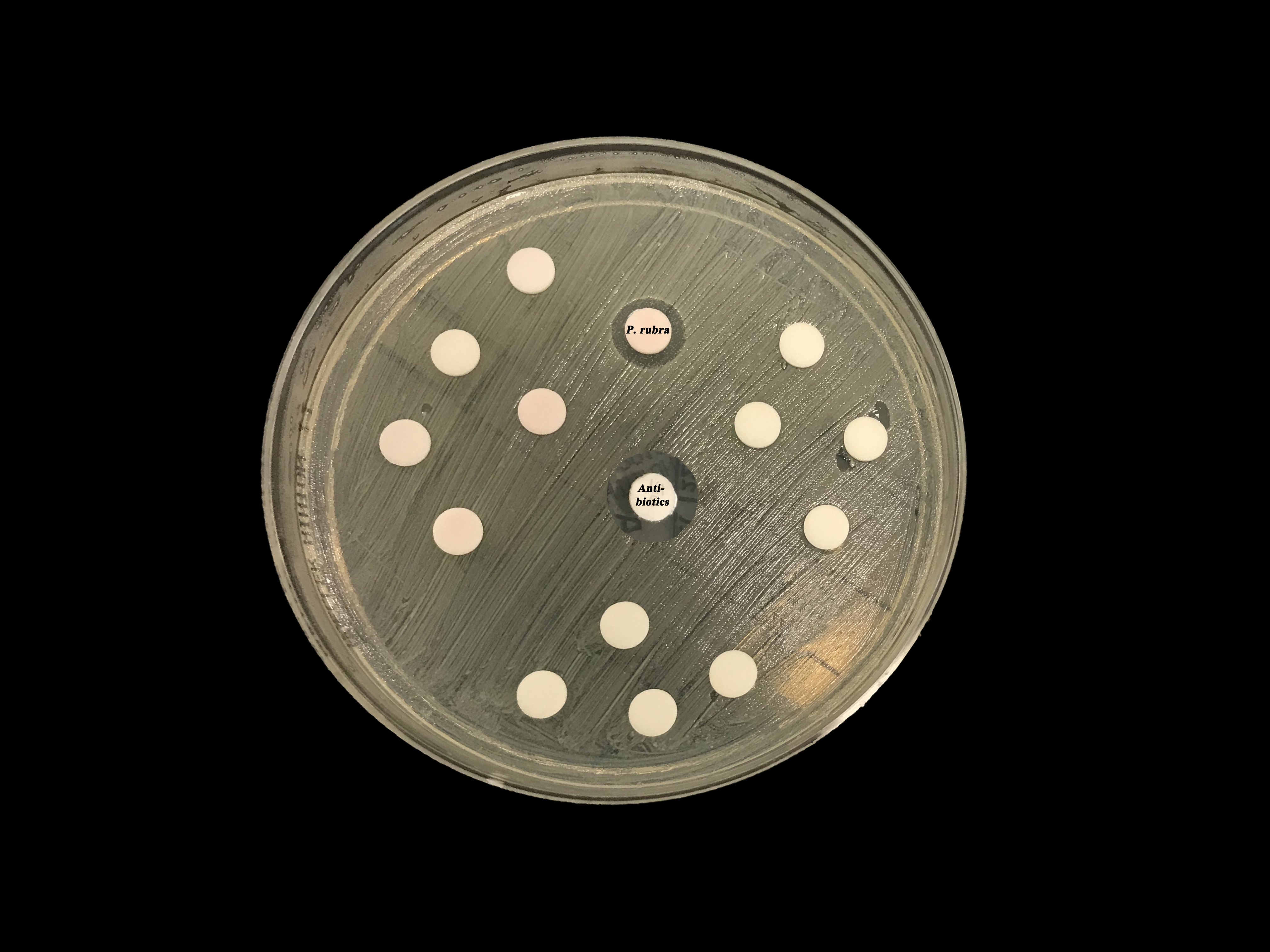

Supplement: Supplemental Information 4 [file peerj-08-8093-s004.jpg]

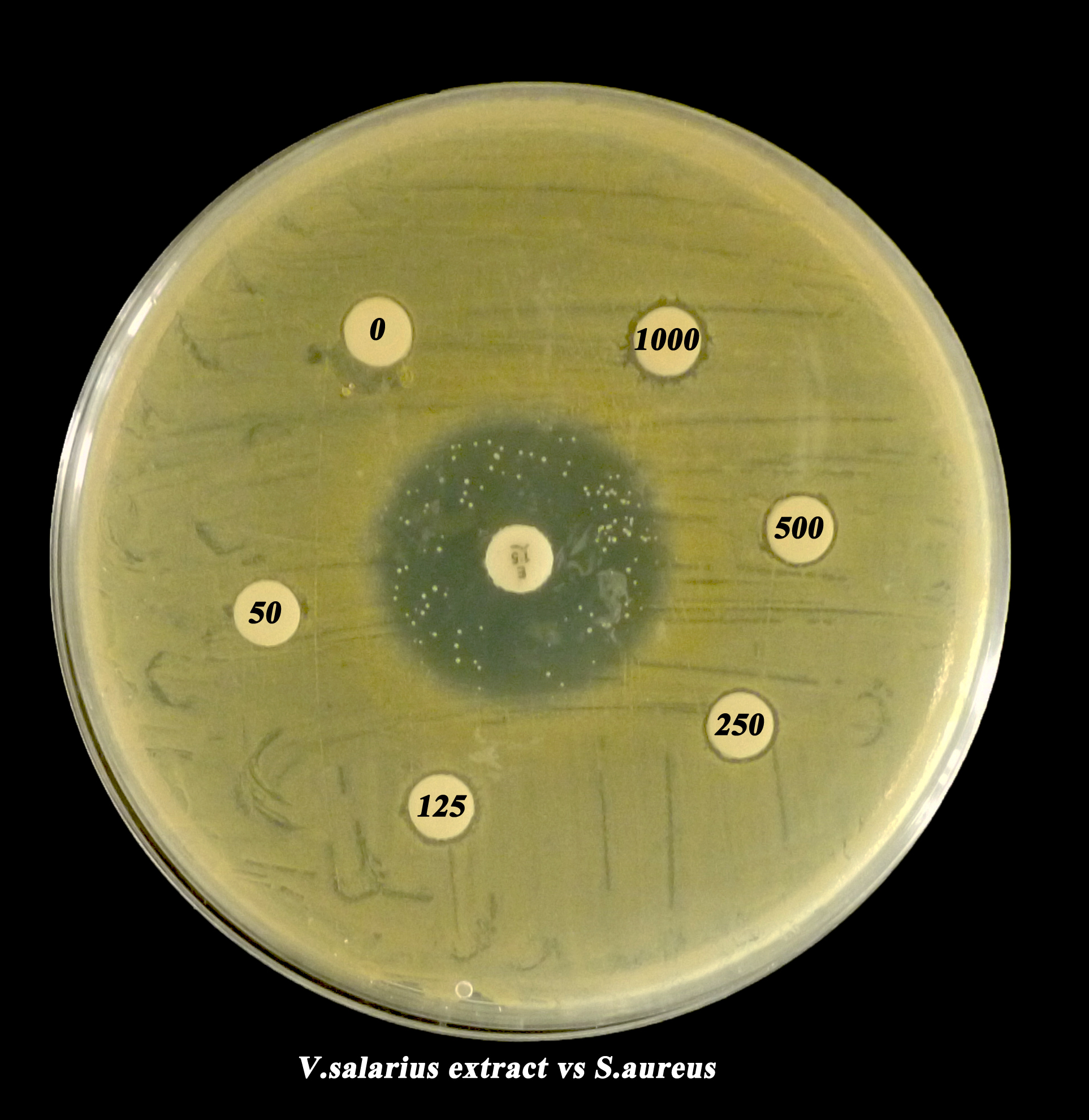

Supplement: Supplemental Information 5 [file peerj-08-8093-s005.jpg]

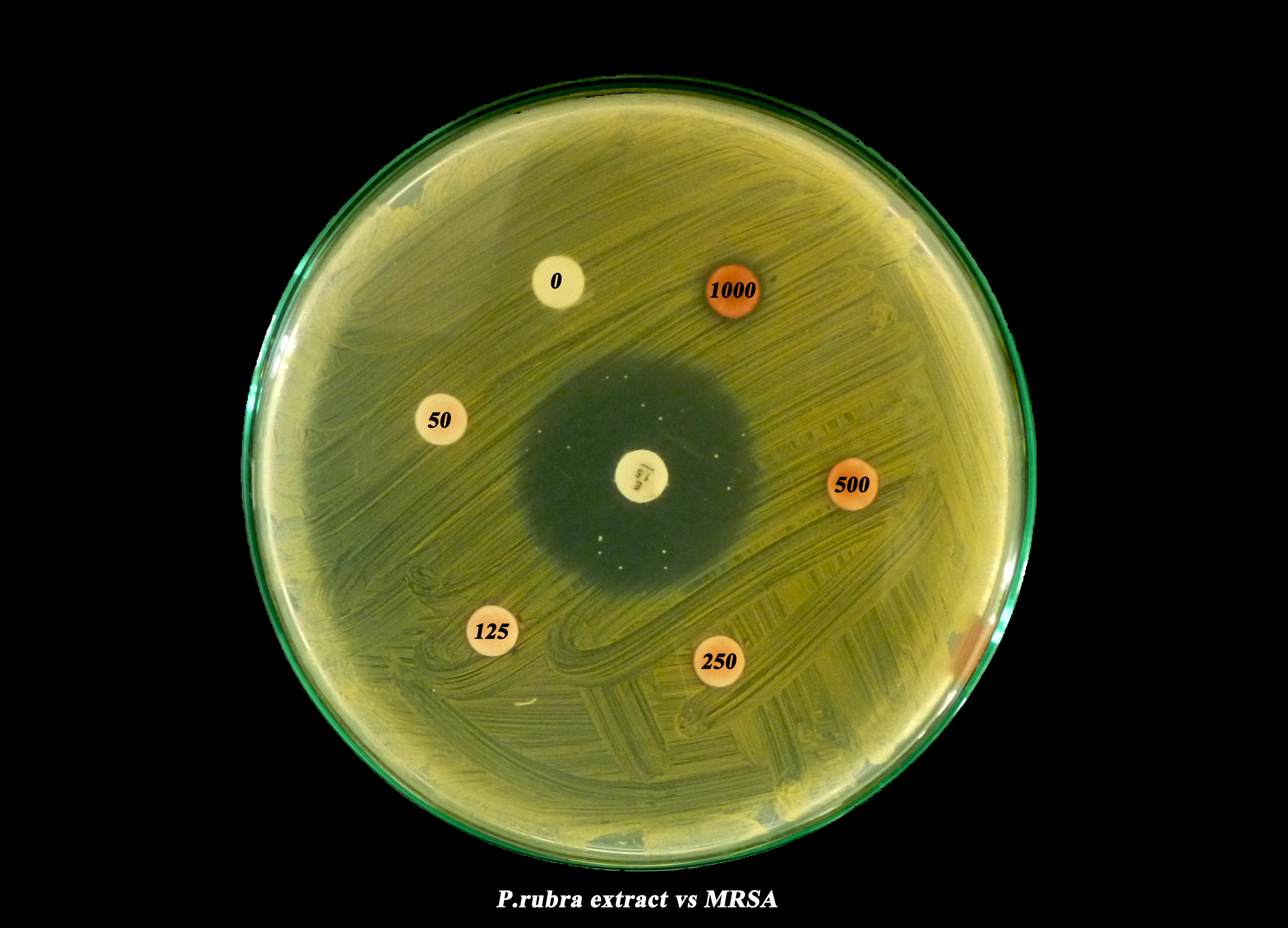

Supplement: Supplemental Information 6 [file peerj-08-8093-s006.jpg]

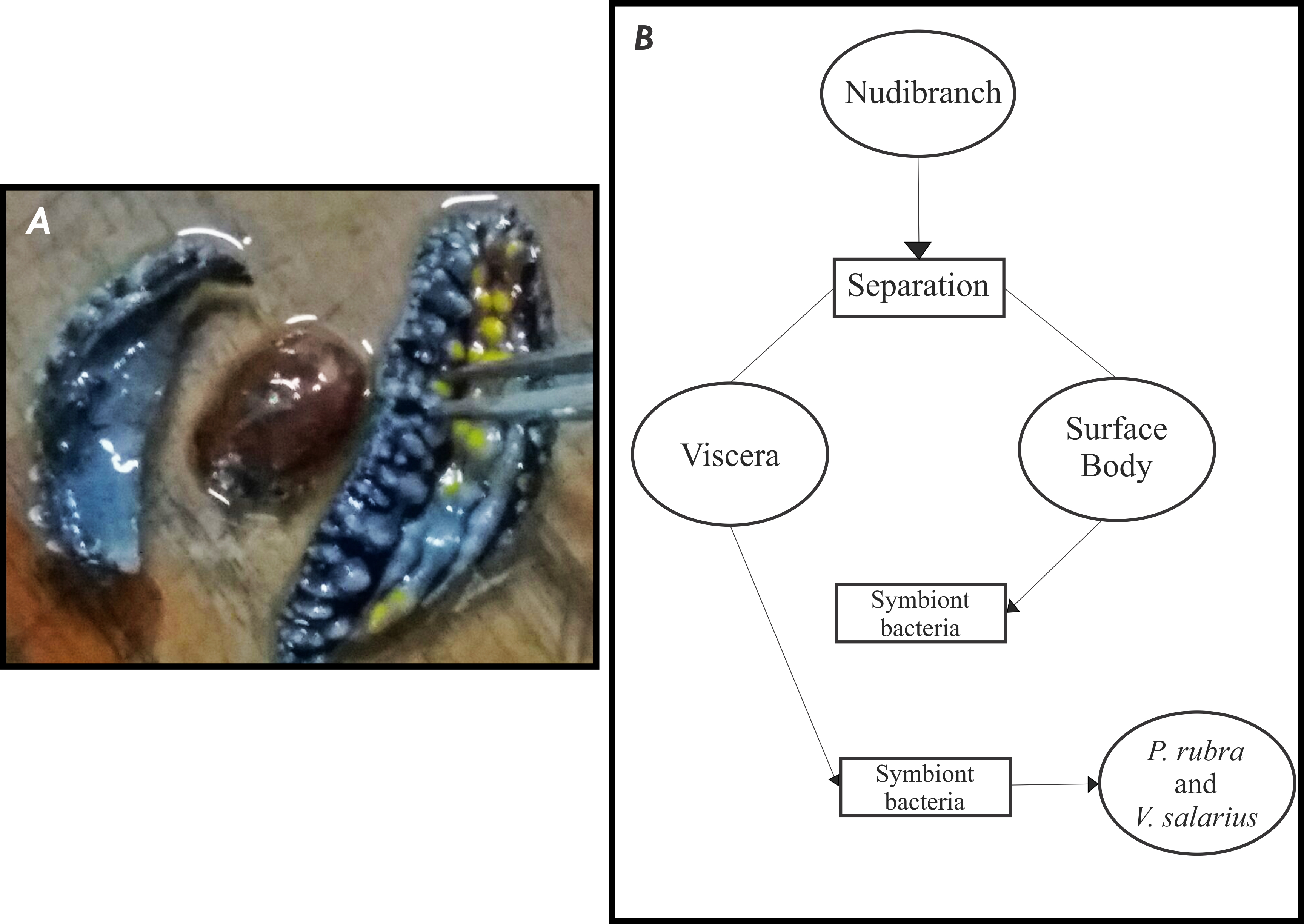

Supplement: Supplemental Information 9 [file peerj-08-8093-s009.jpg]
